# Social Interactions in Zoo-Housed Elephants: Factors Affecting Social Relationships

**DOI:** 10.3390/ani9100747

**Published:** 2019-09-29

**Authors:** Ellen Williams, Anne Carter, Carol Hall, Samantha Bremner-Harrison

**Affiliations:** School of Animal, Rural and Environmental Sciences, Nottingham Trent University, Brackenhurst Campus, Southwell, Nottinghamshire NG25 0QF, UK; anne.carter@ntu.ac.uk (A.C.); carol.hall@ntu.ac.uk (C.H.); samantha.bremnerharrison@ntu.ac.uk (S.B.-H.)

**Keywords:** zoo elephants, social behaviour, welfare, social relationships

## Abstract

**Simple Summary:**

In the wild, elephants live in large, complex social groups. Herds consist of a mixed structure of related females and their calves. One area of concern regarding the maintenance of zoo elephants has been the inability to provide them with social groupings that reflect wild group structure, and whether this impacts on their welfare. Here, we investigated whether a number of factors at the individual (e.g., personality, age or relatedness to others) and zoo (e.g., herd size, presence of calves in the group) level affected the frequency of social interactions in zoo elephant herds. Interactions were defined as positive or negative and then subdivided into physical and non-physical interactions. Social interactions were found to be related to age, personality, presence of calves in the herd, relatedness to other elephants in the herd and species (African or Asian). Calves engaged in the greatest amount of positive interactions but no extreme aggression was observed between any individuals, which was considered indicative of good social management. Increasing understanding about social structures that are affecting elephant relationships enables targeted management plans to be created, in order to provide elephants with the most appropriate social environments. These findings support the recommendations that elephants should be housed in related herds with multiple ages wherever possible, but they also highlight that unrelated elephants can still form compatible and successful social groups.

**Abstract:**

Elephants have complex social systems that are predominantly driven by ecological factors in situ. Within zoos, elephants are held in relatively static social groups and the factors observed driving social relationships in the wild are largely absent. Little research has investigated the effect of social group factors in zoos on elephant social interactions. The aim of this research was to establish whether there is a relationship between social group factors and social behaviour, in order to identify factors that make elephant herds more or less likely to be compatible. Results will facilitate recommendations for optimum social groupings for zoo elephants. Behavioural data quantifying social interactions were collected between January 2016 and February 2017 at seven UK and Irish zoos and safari parks from 10 African and 22 Asian elephants. Social interactions were split into four categories: positive physical, positive non-physical, negative physical and negative non-physical. Social interactions were related to age (positive physical higher and negative non-physical lower in calves than adults), personality (elephants with higher sociability scores engaged in more positive interactions and less negative interactions), presence of calves in the herd (herds with calves had more positive non-physical), relatedness to other elephants in the herd (positive non-physical were higher when relatives were in the group and negative non-physical were higher between unrelated elephants) and species (Asian elephants engaged in more positive non-physical than African elephants). A greater understanding of factors that may contribute to the success of zoo-elephant social groups is important for individual and herd welfare as it will enable evidence-based decisions which have minimal impact on social structures to be executed. This knowledge will enable proactive management approaches to be undertaken and will thus be paramount in ensuring optimal welfare for elephant herds moving forwards.

## 1. Introduction

Elephants have one of the most advanced mammalian social systems [1]. In situ, they live in complex fission–fusion societies [2,3] and display strong affiliative behaviours. The main driving force behind wild elephant social structures and herd dynamics are ecological factors, such as availability of resources and risk of predation [4,5]. Wild elephants predominantly live in related groups of varying sizes; however, researchers have observed behavioural flexibility and adaptability in social groups [6,7,8]. Within zoos, elephants are held in relatively static social groups and the ecological drivers which may dictate wild elephant social group structures are absent or controlled (e.g., dispersal for mating opportunities). Yet it is only recently that researchers have begun focussing on advancing knowledge of zoo elephant social relationships (e.g., [9,10,11]), and no research to date has identified social mechanisms which may be affecting these social relationships.

A range of factors can affect the success of social groups in zoo environments (reviewed in Williams et al. [12]), where environments are typically more static than animals experience in the wild. These include but are not limited to choice of social partners [13,14], past individual experiences [15,16], group size and composition [17], position in the social hierarchy [18], individual compatibility [19] and personality [20]. Understanding social interactions [21] and how social group factors impact social relationships, has ramifications for animal welfare in zoos on both an individual and a group scale [22]. Social complexity, in terms of conspecific (group size and composition) or species (e.g., mixed species exhibits) composition, is an important area of enrichment [23], and has been recognised in elephants as the single most important component to ‘get right’ [24]. Elephant keepers and researchers have highlighted the importance of providing elephants with compatible groups, comprising a range of ages and access to others at night [25]. Historically, however, lack of provision of appropriate social groups for zoo elephants has been highlighted as an area of concern [26,27].

Current research and elephant management guidelines suggest that, wherever possible, elephants should be housed in related, multigenerational family herds [11,25,28,29,30,31]. There are a number of examples where resemblance to wild-type social groups has led to successful social housing in zoo animals. In cotton-top tamarins, resemblance to wild-type groups led to increased breeding success; high infant survival and low incidences of abortion, stillbirth and parental neglect [32]. Moreover, providing chimpanzees with the opportunity to engage in fission–fusion dynamics akin to wild-type interactions led to low aggression rates and reduced aggressive interactions [33], however there is controversy surrounding using the wild as an optimum standard [34,35] and housing zoo animals in wild-type social groups is difficult to do in some species [12]. For example, in large species, such as elephants, replicating wild-type social groups can be logistically difficult, and requirements are likely to vary according to individual circumstances [36]. Therefore, identifying the elements of the wild-type social group that animals require for good welfare within zoos is of paramount importance.

There may be multiple factors which are affecting the success of social groups in zoo animals. Kinship predicts social compatibility in laboratory-housed mice and primates [37] and it is an important predictor of social relationships in wild African elephants [38,39]. However, kinship is not the sole driver in all social interaction networks. Agonistic social networks in ring-tailed coatis are not affected by kinship [40] and female rhesus macaques maintain stable relationships with non-kin social partners [41]. Indeed, social relationships in adult zoo chimpanzees and bottle-nose dolphin calves are also affected by a number of factors. Chimpanzee social relationships are affected by kinship, sex combinations, age differences, time spent together and personality [42,43] whilst bottle-nose dolphin calf companion choices are more driven by calf age, personality and conspecific age than relatedness [44]. In wild elephants, relationships need not be based on kinship [45]. Unrelated reintroduced elephants in Thailand formed successful social groups upon release [8], elephants from heavily poached areas join unrelated herds [6] and an orphaned female who was captive-reared before being released successfully joined a wild herd upon release [46]. Furthermore, in zoo-housed Asian elephants unrelated individuals have developed ‘special relationships’ with others [47,48].

Regular monitoring of social behaviour in elephant dyads can provide valuable insight into group dynamics and has the potential to be important in zoo elephant management [11]. Identification of factors that are most likely to increase the occurrence of positive social interactions and therefore identify potentially socially compatible partners or group size/age compositions would contribute to individual elephant management plans as required by Secretary of States Standards of Modern Zoo Practice (SSSMZP) elephant management guidelines [49]. In order to provide zoo elephants with social groups that optimise their welfare, it is important to first understand factors that affect social interactions. Despite extensive knowledge of social relationships in wild elephants, relatively little research has investigated social interactions in zoo elephants. The aim of this research was to enhance understanding of social interactions in zoo-elephants in the UK and Ireland through analysis of social interactions. Specifically, it was to establish whether there is a relationship between social group factors and prosocial behaviour, in order to try to identify factors that make elephant herds more or less likely to be compatible, and to provide preliminary insight into optimum social grouping.

## 2. Methods

### 2.1. Ethics Statement

All research protocols were approved by the Nottingham Trent University School of Animal, Rural and Environmental Sciences School Ethics Group (reference number ARE188). Permission to conduct the study was granted by the participating zoos prior to commencement of data collection. Support for the study was obtained from the British and Irish Association of Zoos and Aquariums (BIAZA) Research Group.

### 2.2. Subjects and Study Sites

Subjects were 10 African (1 male: 9 females) and 22 Asian (3 male: 19 female) elephants housed at 7 zoos and safari parks in the UK and Ireland. Herd size ranged from 2 to 9. An additional male Asian elephant housed at Zoo E could not be included in the data set due to missing data (Table 1).

### 2.3. Data Collection

Data collection followed the same methods as detailed in Williams et al. [50]. For completeness, protocols are described in brief below. Elephants were identified using visually discernible differences: height, size and shape of ears, length of tail and presence/absence of hair, scars and tattoos. Data were recorded via live and video observations. Live observations were conducted from public viewing areas during zoo visitor hours. Video footage was either provided by the study zoo from existing cameras (Zoo A, C and E), or cameras were temporarily installed on site (Zoo D, F and G). Where cameras were installed, video recordings were made of outdoor enclosures using high definition video cameras with infrared capability (Hikvision IR network camera, Model DS-2CD2632D-IS, Hikvision Europe B.V., Hoofddorp The Netherlands). Cameras had a 20 m IR light range and recorded at 20FPS onto bespoke recording kits designed by Carnyx Wild (Carnyx Wild, Skipton, UK). To comply with data protection laws, no sound recordings were made [50].

The main data collection period ran from January 2016 to February 2017 (Zoo A, C, D, F: January, February, April, May, July, August, October, November 2016; Zoo B: May, August, December 2016, February 2017; Zoo E: February, April, May, September, October, November 2016; Zoo G: January, February, April, May, July, August, September, November 2016). Observations were undertaken by a single observer. Data were collected over a 5-day period each month with each 24 h day split into 12 × 2-h periods. Within each 2-h period, data were collected for 1 h. Observations were stopped whenever elephants were involved in keeper-initiated interactions (e.g., public feeding displays or training). There was a discrepancy in the hours of observations which were able to be undertaken across the study zoos due to external circumstances, e.g., failure of recording equipment, and it not always being possible to view all study elephants for the full duration of each observation period due to enclosure set-ups. Data were therefore analysed as a proportion of total possible observations, to enable cross-zoo comparisons to be made.

### 2.4. Social Interactions

Scan sampling and instantaneous recording with a 30-s inter-scan interval was employed to reduce sampling bias, e.g., only recording the first elephant to take part in an interaction, or to limit introducing an error in interpretation of the context of the interaction. Social interactions were split into positive and negative interactions. Interactions were considered to be positive if they were non-aggressive contact or non-aggressive approaches (e.g., touching with the trunk), and negative if they were instances of aggression or a reaction to aggressive behaviour (e.g., walking away from another elephant) [31,47]. Positive and negative social interactions were subdivided into physical and non-physical interactions (Table 2) [50].

### 2.5. Factors Affecting Social Interactions

A number of social group factors (age of elephants (years), relatedness to others, species, origin, sex, study zoo and personality) were investigated to determine their relationship with elephant social behaviour. Details pertaining to individual elephants (age, relatedness to others etc.) were gathered from Species 360 Zoological Information Management System (ZIMS). Development of the elephant personality assessment has been described in Williams et al. [50], however a brief overview is provided here. An elephant personality assessment questionnaire comprising 21 adjectives (Table 3) was distributed to study zoos. Ratings were made on a 10 cm visual analogue scale with the anchors ‘disagree’ (0 cm) and ‘strongly agree’ (10 cm). Exact scores were determined by measuring the distance (in centimetres, to 1 dp) along the line that the rating was placed.

### 2.6. Statistical Analysis

Data were expressed as a proportion of time elephants could have been observed within the observation period to prevent over-representation of sociability. Analysis of social interactions focused on frequency of time spent ‘giving’ social interactions rather than ‘receiving’ so as to gauge how socially active each individual was as opposed to measuring their popularity.

Statistical analysis for keeper ratings of personality and the difference between social interactions across age categories was undertaken in SPSS version 21 (SPSS Inc., Chicago, IL, USA). Full details of the personality analysis are reported in Williams et al. [50]. Intra-class correlation coefficients (ICC (3,k)) were calculated for each personality adjective to determine inter-rater reliability. Adjectives with an average ICC of <0.5 were removed from further analysis. A principal components analysis (PCA) was conducted to reduce the remaining personality adjectives into components. The component solution was rotated using varimax rotation and components with eigenvalues >1 were extracted. Adjectives with salient loadings (>0.4) on more than one component were assigned to the component on which it had the higher loading. Cronbach’s alpha was used to detect internal consistency. Composite scores were calculated as the mean of the adjectives within each component. A Wilcoxon signed rank test was undertaken to identify whether there were differences between the frequency of positive and negative social interactions. A Kruskal–Wallis test was used to investigate frequency of social interactions across age categories. Elephant age was split into the following categories for data analysis: calves (0 to 2 years), infants (3 to 4 years), juveniles (5 to 9 years), sub-adults (10 to 15 years) and adults (16 years and older) [51].

General linear models (GLMs) were used to investigate the influence of the reviewed factors on proportion of time individuals spent giving social interactions to the rest of the herd. Proportion of positive and negative physical interactions and positive and negative non-physical interactions were fitted as response variables, following quasibinomial error structures. Factors were fitted as separate fixed effects. Due to sample size limitations, models were simplified and fixed effects were tested individually. All data analysis for GLMs was undertaken in R (Version 1.1.383) (Boston, MA, USA) using package lme4. Model results are reported as model estimate (β_1_) ± SE.

## 3. Results

### 3.1. Social Interactions Overview

**Figure 1:**
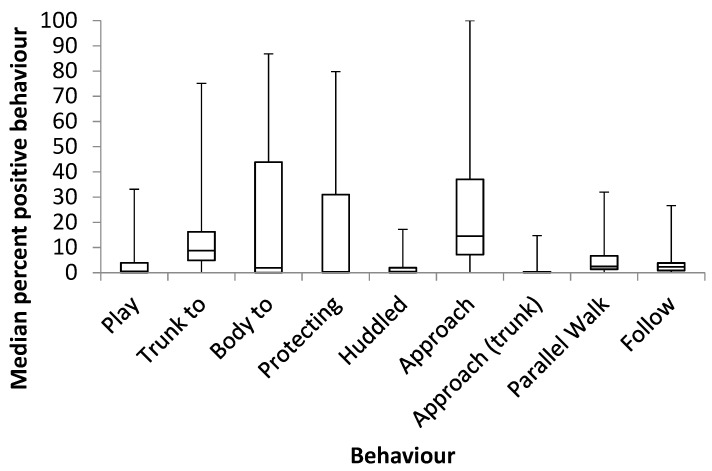
A breakdown of positive interactions observed. Conspecific play, trunk to- and body to- were grouped as physical interactions.

**Figure 2:**
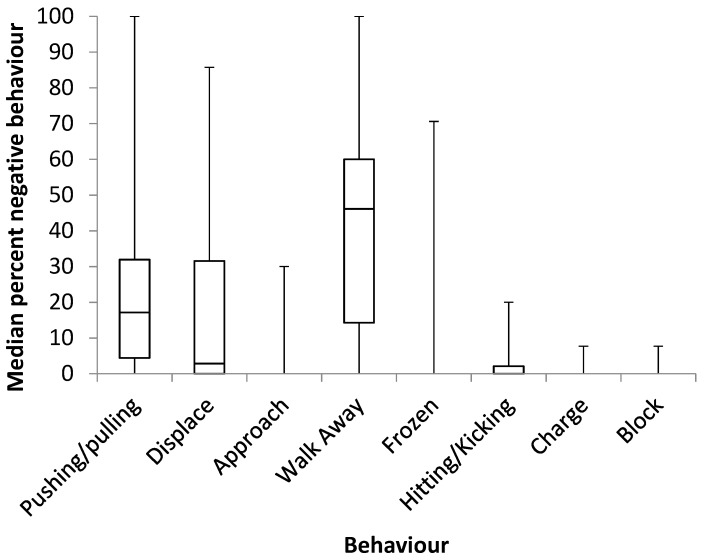
A breakdown of negative interactions observed. Pushing/pulling and hitting/kicking were grouped as physical interactions.

Elephants (*n* = 32) engaged in more positive interactions than negative interactions (positive physical (median, IQR): 4.33% (0.48–24.50), negative physical: 0.09% (0.04–0.19), positive non-physical: 8.46% (3.31–17.51), negative non-physical: 0.46% (0.21–1.17) (χ^2^ (3) = 62.687, *p* < 0.001). Positive physical interactions were more frequent than negative physical interactions (Z = −4.623, *p* < 0.001) and negative non-physical interactions (Z = −3.606, *p* < 0.001). Positive non-physical interactions were more frequent than negative physical (Z = −4.860, *p* < 0.001) and negative non-physical interactions (Z = −4.742, *p* < 0.001). Negative non-physical interactions were more frequent than negative physical interactions (Z = 4.644, *p* < 0.001). A breakdown of types of positive interactions is provided in Figure 1. ‘Conspecific play’, ’trunk to-‘ and ‘body to-‘ were grouped as physical interactions. Trunk to- interactions were the most frequently occurring positive physical interactions, accounting for median 9.6% (IQR: 4.5–17.5%) of all positive interactions (range 0–75%). In total, 88% of the study elephants engaged in positive trunk to- behaviours, whereas only 9% engaged in negative trunk to- behaviours. A breakdown of types of negative interactions is provided in Figure 2. Pushing/pulling and hitting/kicking were grouped as physical interactions.

### 3.2. Personality Assessment

Full results of the keeper ratings of personality are provided in Williams et al. [50], however for clarity information which is needed in order to be able to interpret data generated from this study is provided here. Keeper assessments of personality were completed by 27 keepers across the seven study zoos. Nine personality adjectives were reliably rated and thus entered into a PCA to identify personality components. The PCA revealed three components: ‘calm’, ‘sociable’; and ‘engaged with the environment’ [50]. The data included in this paper pertain only to the sociable personality component. The following adjectives loaded positively onto the sociable personality component: sociable (0.925), affectionate to elephants (0.878) and playful with conspecifics (0.697). No adjectives loaded negatively onto the component. Cronbach’s alpha revealed good internal consistency (0.857).

### 3.3. Positive Social Interactions

**Figure 3:**
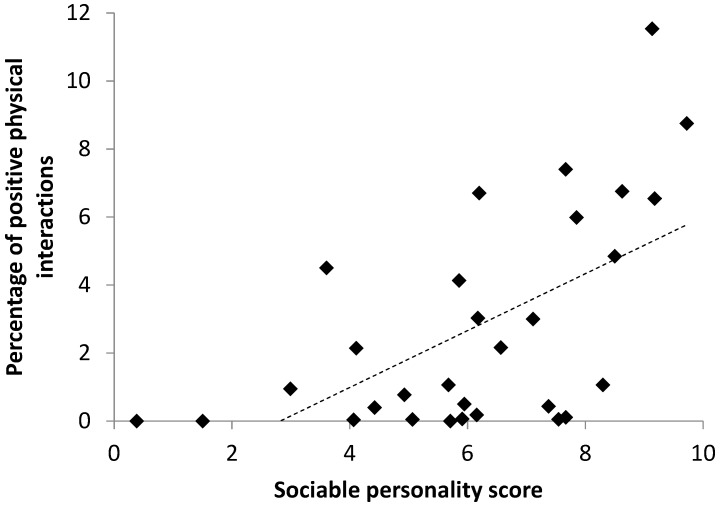
Relationship between sociable personality component score (assigned by keepers) and positive physical interactions given.

There was a positive correlation between positive physical social interaction frequency and the sociable personality component score (β_1_ ± SE; 0.41 ± 0.11, *t* = 3.861, *p* < 0.001) (Figure 3). There was a negative relationship between age and frequency of physical social interactions (−4.15 ± 0.29, *t* = −14.281, *p* < 0.001). Calves engaged in four times more positive physical social interactions than adults (χ^2^ = 11.952, *p* < 0.01) (Table 1). There was no effect of relatedness to others, origin, zoo, sex, species, herd size or whether or not a calf was present in the herd on positive physical social interaction frequency.

There was a relationship between positive non-physical interactions and relatedness to others, species and the presence of a calf in the herd. Positive non-physical interactions were on average three times higher when elephants had a relative in the group than when they did not (1.08 ± 0.38, *t* = 2.803, *p* < 0.01) and they were three times lower when calves were not present (−1.29 ± 0.37, *t* = −3.488, *p* < 0.01). Interactions between related individuals were on average three times more frequent than between unrelated individuals (Table 4). Positive non-physical interactions were higher in Asian herds than African herds (1.15 ± 0.45, *t* = 2.58, *p* < 0.05). There was no relationship between the sociable personality component, elephant origin, zoo, age, sex or herd size and positive non-physical interactions.

### 3.4. Negative Interactions

None of the investigated factors (age of elephants, relatedness to others, species, origin, sex, study zoo and personality) were correlated with negative physical interactions (*p* > 0.05). Negative non-physical interactions were affected by age (−2.27 ± 1.08, *t* = −2.105, *p* < 0.05); calves engaged in ten times fewer negative non-physical interactions than adults (χ^2^ = −16.800, *p* < 0.01). There was also a negative correlation between herd size and negative non-physical interactions (−0.20 ± 0.08, *t* = 2.473, *p* < 0.05) and the degree to which they were considered ‘sociable’ by keepers (−0.25 ± 0.05, *t* = −4.664, *p* < 0.001). Unrelated elephants engaged in three times more negative non-physical interactions than related elephants (−0.77 ± 0.33, *t* = −2.313, *p* < 0.05). There was no relationship between zoo, the presence of a calf, species, sex or origin and negative non-physical interactions.

## 4. Discussion

A number of the investigated social group factors affected social interactions in the observed herds. These findings contribute important knowledge to a relatively unknown subject area. No overt aggression was observed during the study, with only minimal occurrences of ‘correctional’ behaviours such as trunk slap and kicking [51] recorded. This finding may be due to management of social incompatibilities by the study zoos to minimise the occurrence of excessive aggression (Cairns, personal communication, 2016). Elephant keepers describe low levels of aggression as ‘completely normal’, however escalating aggression can be a cause for concern [25], and typically results in intervention by the zoo to prevent ongoing occurrences (Cairns A., personal communication, 2016). Types of social behaviour recorded in this study, such as touching with the trunk, conspecific play, approaching conspecifics and displacement, were similar in nature to reports in other studies of zoo elephants [10,11,52,53,54,55,56,57]. Positive non-physical interactions were higher in Asian herds than African herds, however Asian elephants were held, on average, in, larger (mean herd size 5.5 Asian, 3.3 African) and more related herds and so it is not possible to decipher from the data whether this finding is due to relatedness or to species.

### 4.1. Age and Oresence of Calves

Positive physical interactions in this study were predominantly categorised as trunk to- behaviours (touching another elephant with the trunk in a non-aggressive manner) or social play. Trunk to- behaviours are a means of providing reassurance and comfort in elephants [58]. Positive interactions and specifically conspecific play were related to the presence of calves in groups and age of individuals, which was predicted based on knowledge of wild elephant social group structures. The majority of conspecific play was observed at zoos which had calves in the herd and the highest frequency was recorded between bull elephant calves. Care of offspring is a pivotal component in elephant social structure [59] and reintroduced elephants form groups associated with the presence of an elephant calf, leading researchers to call for reintroductions to include groups of calves or adults with calves to increase the chance of successful group formation and long-term establishment of stable herds in situ [8]. The limited field of zoo research has also found that social interactions in zoo elephants are centralised around the presence of calves. Calves engage in most social interactions [47] and connect groups through initiation of social interactions [9]. Research in gorilla groups suggests that formation of new social groups is most likely to be successful when individuals are young [60]. In wild African elephants, the most frequent interaction type between immature elephants, especially young bulls, was social play [60].

The decrease in social interactions given by older elephants, however, is an interesting finding and one which could have a number of potential explanations. It could be that older elephants may have different backgrounds (e.g., wild caught; 44% of the study elephants were wild caught, all of these were ‘adults’) and they could have experienced different early management. Research has shown that elephants reared in social isolation may have impaired development [61] and thus may not know how to interact socially, so if elephants have spent time in isolation in previous years this may have affected their social development. However, it could also be that older elephants do not need to perform physical reassuring behaviours as frequently as calves. The latter theory is supported by the lack of significant relationship between origin of elephants and frequency of social interactions in this study. Elephant calves develop at a faster rate when they are exposed to physical contact [62] and touch in elephant calves plays a role in normal development as well as enabling young elephants to test their strengths and capabilities with one another [52,63,64]. Calves are described as the herd nucleus in elephant groups [9,65] and in the wild the allomothering of calves works to increase both calf survival and group stability [63]. Touching (or trunk to-) behaviour could be a reinforcement of the social bond in the direction of older female to calf or it could be a result of a need for reassurance from the calf. It could also represent a change in social interactions as individuals grow older. Wild adult elephants do engage in elaborate greeting rituals following separations, even if separation lasts for only a few minutes [66]. However, separations, especially for long periods of time, were not present in the study period. Further research should incorporate behavioural response to routine separation as a measure of social bond strength in adult elephants. The concept of social behavioural change as individuals age has been reviewed in Krebs et al. [67], however it is important to be able to separate a gradual change in social engagement as a result of natural aging from more serious health and therefore welfare problems.

Harris et al. [27] advocated the need for herd structures with a range of ages, and these findings support this notion, but only for breeding herds, to provide companionship for youngest elephants and appropriate opportunities for social learning as they develop. However, an absence of calves in a herd does not necessarily lead to poor welfare. A lack of elephant calves did not lead to a lack of interactions within the study herds; adults were observed engaging in positive physical interactions with one another when calves were not present. Investigating association rates in terms of proximity to others may be important for herds with older members, where relationship strength may be better assessed using association data rather than physical interactions only.

### 4.2. Relatedness to Others

Lack of relatedness to other elephants in herds is one of many concerns for zoo elephant welfare, and researchers have suggested it can lead to aggressive behaviours [26,68]. In this study, non-physical interactions were extremely rare (median 0.09% activity) and no overt aggression was observed. The most frequently observed negative physical interactions were pushing/pulling and hitting/kicking, which have been described as ‘short-term disciplinary behaviours’ [51]. Positive physical interactions were not affected by relatedness to others, but the frequency of positive non-physical interactions were higher and negative non-physical interactions were lower in elephants who had at least one relative in the herd. Historic reasons for limiting social choices and chaining/tethering elephants overnight were that there may be aggression between individuals [53,69]. The lack of overt aggression observed during this study suggests that this concern, in the UK and Ireland, is unfounded. Similar findings have been reported in other studies of zoo-elephant social behaviour, when physical aggression accounted for 0.5% or less of observed behaviour [70,71,72] and unrestricted access to others had no negative effects on behavioural profiles [53].

Low levels of aggression are described by keepers as ‘normal’, an integral part of maintaining the hierarchical herd structure [25]. Zoo management guidelines suggest that, where possible elephants should be kept in related, matriarchal family herds [28,29]. However, there is a need to house a number of unrelated elephants within UK and Irish zoos who are already part of the zoo population, and this trend is likely to continue whilst elephants are brought in from circuses or other zoos in Europe or are moved as part of European breeding programmes. Genetic relatedness predicts fission and fusion of social groups in wild African elephants, and associations between social groups persist long after original maternal kin have passed away [73] but genetic relatedness is not the sole driver of wild elephant social relationships [6,8,45,46]. In zoo-housed chimpanzees, time spent together is one factor affecting social relationships between individuals [42,43]. It was not possible to look at years spent together for the study elephants in a measurable way as it was not always clear how long individuals had spent together prior to coming to the study zoos, for example, some had been housed together in previous collections. However, it could be possible that within zoo herds familiarity is as important as relatedness in individual compatibility and this is an area which should be investigated more thoroughly in the future.

### 4.3. Species Differences

Positive non-physical social interactions were more frequent in Asian elephants than African elephants, although there were no species level differences for positive physical, negative physical or negative non-physical interactions. The reason for these differences is unclear but it is possible that they are the result of a lack of equality in the observed social groups in terms of age structure and relatedness. Generally, Asian elephants were kept in larger and more related groups than African elephants in the study, and none of the studied African elephant herds had calves in the groups. African and Asian elephants are presently treated as one species in terms of management guidelines [49]. In the wild they have different social structures; African elephants predominantly live in larger and more complex social groups than Asian elephants [3], although both species have strong social bonds within their social groups [2,3,74,75]. Differences in social structure in wild African and Asian elephants relate to the size and complexity of social groups; African elephant social groups are generally larger [75] and more connected than wild Asian elephant social groups [3]. These structural differences are likely an influence of their wild environments and thus may not be so prevalent in zoos. It is extremely important to consider species level differences in future studies of elephant social structures in zoos; if there are biologically relevant species-level differences in their social structures within zoos, which replicate wild-type differences, then consideration should be given to developing species specific guidelines, in order to ensure optimal welfare for all individuals. It may be that greater consideration should be given not just to species-level interactions, but also to group type, e.g., family group, bachelor herd or unrelated non-breeding females, to ensure all individual needs are being met within the social group.

### 4.4. Personality

Sociable personality component scores were related positively to positive physical interaction frequency and negatively to negative non-physical interaction frequency. Thus, keeper ratings of elephant sociability predict prosocial behaviour. This is potentially extremely important in elephant management as it highlights the possibility of using keeper ratings as a proxy for behavioural observations [50]. Understanding more about the relationship between personality and friendship choices in elephants may be important for both current and future welfare of zoo elephants. If personality enables a means of assessing social compatibility in elephants it could help to predict the potential for social compatibility between elephants in future moves.

### 4.5. Factors Not Related to Social Interactions

Not all of the investigated factors were related to social interaction frequency. There was no relationship between social interactions (physical or non-physical) and elephant origin, which suggests that neither being born into a zoo nor coming from the wild predicts the ability of individuals to exist in a functional social group, a very important and promising finding for zoo elephants. It suggests that if provided with an appropriate social environment there is the potential to maintain good welfare for all zoo elephants, regardless of prior experiences or birth place. There was also no behavioural difference between male and female elephants although this finding could be affected by the low number of adult bulls observed. In the wild, there is a great deal of variation in terms of behavioural development in bulls and cows [76] and this should be borne in mind when providing bull elephants with social companions in zoos. Only two adult bulls were observed during the study and these were only able to be recorded during daytime hours in outside enclosures. All other males were calves. One potential limiting factor in enabling long-term success of social groups is prevention of opportunities to independently form new families. This is particularly prevalent for young bull elephants who would usually separate from their family groups during adolescence [2]. Elephant management guidelines are relatively lacking in terms of the needs of bull elephants [77]. It is therefore advocated that further research is undertaken to explore potential differences between the social needs of male and female elephants.

There was no relationship between positive physical interactions and relatedness to others, origin, zoo, sex, species, herd size or presence of a calf in the herd, and no relationships were observed between negative physical interactions and any of the investigated factors. The lack of evidence to support a link between group size and prosocial behaviour lends empirical evidence to support arguments made by elephant keepers and researchers that, within reason, compatibility of elephants is of greater importance than a minimum group size [25]. These findings may, however, be due to the low frequency of physical interactions recorded during the study. Further investigation of elephant sociability in terms of association data may reveal more relationships with the investigated factors and lend further support to these initial important findings.

It is likely that it is a combination of multiple, interacting factors that are affecting the success of elephant social groups, and it is important to recognise that the structure of social groups can change over time and should be managed accordingly. Stability of elephant social groups in terms of group members can limit long-term success in zoo elephant social groups. Social groups may be subject to enforced change (e.g., due to limited space or gender of offspring) and they do not have the opportunity to independently form new social units. Understanding factors that may affect zoo elephant social relationships can help to alleviate social pressures by predicting social compatibility, or at least to identify ‘risk factors’ which may reduce the likelihood of compatibility. However, it is recommended that further research is undertaken to monitor change in relationships over time, especially as young animals develop or when individuals are moved to create new herds. Future work should also seek to investigate the relationship between physical interactions, proximity to other elephants and measures of a number of indicators of welfare, to further understand the relationship between physical interactions, proximity and physical and physiological welfare. Establishing a greater understanding of herd dynamics and the role of social behaviour in elephant welfare will support evidence-based management which will help to optimise welfare. Methodologies used in this study have applicability in other socially housed zoo species, determining social requirements in order to provide good welfare.

## 5. Conclusions

Appropriate social groups comprising compatible individuals can be one of the hardest things to provide social species in zoos, especially an animal with needs as complex as an elephant. Historically, researchers looked to wild elephant social groups to predict zoo elephant social wants and needs, but the zoo environment is artificial and social groups are more fixed than in the wild. Furthermore, the pressures driving social group formation and existence are not present within zoos, and so factors driving social group success in zoos may be different to the wild. The occurrence of positive social interactions has been identified as an important yet understudied indicator of welfare in zoo elephant social groups. Recent research has begun to focus more on social interactions in zoo elephants and current guidelines recognise the importance of individual compatibility. It is likely that a number of factors may affect zoo elephant social relationships and identification of these is important for future welfare. The results from this study show that elephant social interactions are related to age, personality, presence of calves in a herd, relatedness and species. Whilst it is important to recognise that these factors may be to some extent overlapping, this study has made important first steps to identify things that may be affecting the success of zoo elephant relationships. These results must, however, be interpreted with some caution and it is recommended that this preliminary research should be repeated to enable zoo-wide recommendations to be made. The most interconnected group was the largest group with the greatest number of calves, however elephants held in smaller groups also engaged in a range of prosocial behaviour. The lack of a statistically significant link between herd size and positive social behaviour lends evidence to support suggestions by elephant keepers that the recommended minimum group size of four individuals (currently a criterion in the SSSMZP elephant management guidelines) may not be as important as compatibility for individual elephant welfare. The degree to which elephants can be considered sociable is individual. Being able to predict factors that may contribute to the success of social groups is important for individual and herd welfare. Further work is needed to investigate the relationship between the social group factors identified and welfare, to document whether or not there is a direct link between the occurrence of positive or negative social interactions and individual elephant welfare. Taking into account individual life histories and social needs at different life stages is also an important area for consideration. Pro-active management approaches based on increased knowledge of elephant social needs is important in ensuring long-term optimal welfare moving forwards.

## Figures and Tables

**Table 1:** Elephant and herd demographics for the study elephants at the onset of the study period (October 2015).

Zoo	Elephant	Species	Sex	Age	No. Relatives in Herd	Wild or Captive Born	If Zoo Born, at Natal Zoo?	Observation Period (mins)	Proportion Observations in Sight
A	E1	African	F	45	0	Wild	N/A	5817	0.66
	E2	African	F	47	0	Wild	N/A	5817	0.98
B	E3	Asian	F	54	0	Wild	N/A	5842	0.89
	E4	Asian	F	44	0	Wild	N/A	5842	0.89
	E5	Asian	F	40	0	Wild	N/A	5842	0.85
C	E6	Asian	F	50	0	Captive	N	5838	0.75
	E7	Asian	M	15	1	Captive	N	5838	0.16
	E8	Asian	F	1	4	Captive	Y	5838	0.90
	E9	Asian	F	36	3	Wild	N/A	5838	0.78
	E10	Asian	F	19	3	Captive	Y	5838	0.87
	E11	Asian	F	13	3	Captive	Y	5838	0.87
D	E12	African	M	34	0	Wild	N/A	7666	0.20
	E13	African	F	35	0	Wild	N/A	7666	0.27
	E14	African	F	35	0	Wild	N/A	7666	0.67
	E15	African	F	31	0	Wild	N/A	7666	0.69
E	E16	Asian	F	32	8	Captive	N	3267	0.65
	E17	Asian	F	26	8	Captive	N	3267	0.66
	E18	Asian	F	13	8	Captive	N	3267	0.71
	E19	Asian	F	10	8	Captive	Y	3267	0.75
	E20	Asian	M	2	9	Captive	Y	3267	0.61
	E21	Asian	F	2	9	Captive	Y	3267	0.65
	E22	Asian	M	2	9	Captive	Y	3267	0.60
	E23	Asian	F	<1	9	Captive	Y	1569	0.51
	-	Asian	M	22	9	Captive	N	-	-
F	E24	African	F	14	1	Captive	Y	5031	0.79
	E25	African	F	30	0	Wild	N/A	5031	0.76
	E26	African	F	14	2	Captive	Y	5031	0.81
	E27	African	F	30	1	Wild	N/A	5031	0.80
G	E28	Asian	F	33	0	Wild	N/A	5016	0.69
	E29	Asian	F	22	1	Captive	N	5016	0.70
	E30	Asian	F	3	1	Captive	Y	5016	0.63
	E31	Asian	F	19	1	Captive	Y	5016	0.68
	E32	Asian	F	34	1	Wild	N/A	5016	0.67

No social behaviour data was available for the bull elephant at Zoo E due to video camera quality from outside enclosures. He was therefore removed from the study.

**Table 2:** Elephant behaviour ethogram (based on Asher et al. [31]) [50].

Behaviour			Description
Positive	Positive physical	Conspecific play	Engaging in active play with another elephant, including head-to-head sparring, trunk wrestling, mounting, chasing and rolling on one another. Does not include behaviours observed following an agonistic encounter or courtship.
		Touching (trunk to)	Touching another elephant with the trunk in a non-aggressive manner.
		Touching (body to)	Touching/rubbing another elephant with the body.
	Positive non-physical	Protecting	Standing over another elephant.
		Huddling	Formation of a tight circle with calves at the nucleus. Calves hidden in the middle, adults surrounding them.
		Approach	Walking towards another elephant in a non-threatening manner. Recipient stays in position during and after the approach.
		Approach with trunk	Trunk outstretched towards another elephant. Not close enough to make physical contact.
		Walking with	Walking side by side with another elephant.
		Following	Walking closely behind another elephant (within one elephant body length).
Negative	Negative physical	Pushing	One elephant forces or pushes against the body (usually the rump) of another elephant, resulting in the elephant that is being pushed moving at least two steps.
		Pulling	Using the trunk to pull at another elephant in a non-playful manner. May pull at the trunk or an accessible body part such as tusks/tushes or the tail.
		Sparring	An escalation of a push/pull incident into more physical aggression.
		Hitting/kicking	Aggressive physical contact with the trunk or leg, e.g., trunk strike or kicking out.
	Negative non-physical	Displace	Movement of one elephant results in another elephant leaving its location (within 10 s)—usually occurs when a more dominant elephant approaches a more subordinate individual.
		Approach	Walking towards another elephant in an aggressive or hostile manner (head held high, ears wide or flapping). Receiving elephant may either respond to this by standing as tall as possible, head raised, ears flapping or turning away from/walking away from the approaching elephant.
		Walking/turning away from	Avoiding or shying away from elephants or people; the individual either walks forwards away from or backwards away from a particular elephant or person.
		Frozen	Standing still and alert as another elephant approaches.
		Charge/mock charge	Move towards another elephant with the head held high, pace usually quickens as individual gets closer to the target elephant. In the case of a mock charge the individual charging stops further away from the target elephant.
		Blocking	Blocking from food source or other resource (e.g., door).

**Table 3:** Adjective and behavioural definitions included in the elephant personality assessment sent to keepers (*n* = 27) at the study zoos (*n* = 7) to assess the profiles of their elephants (*n* = 30) [50].

Adjective	Definition
Active	Has high motivation to be physically active
Adaptable	Quickly adapts to novel situations
Affectionate (keepers)	Seeks close relationships to keepers
Affectionate (elephants)	Seeks close relationships to elephants (please place two lines if there is a difference for related or un-related elephants)
Aggressive	Causes harm or potential harm to conspecifics, e.g., displays, chases, bites
Apprehensive	Seems anxious; fears or avoids risk
Calm (unfamiliar people)	Reacts to unfamiliar people in a calm and peaceful manner
Calm (novel situations)	Reacts to novel situations in a calm and peaceful manner
Confident	Behaves in a positive, assured manner
Curious	Shows interest in novel objects
Fearful (conspecifics)	Retreats readily from conspecifics
Fearful (disturbances)	Retreats readily from outside disturbances
Inquisitive	Explores new situations and tries to learn new things
Mischievous	Shows a fondness for causing trouble in a playful way, e.g., sand kicking or trunk grabbing
Playful (conspecifics)	Initiates or readily engages in play with conspecifics
Playful (objects)	Readily engages in play with objects
Placid	Reacts to conspecifics in an even, calm way; is not easily disturbed
Restless	Rarely relaxes, always walking or moving around the enclosure
Sociable	seeks companionship of conspecifics
Solitary	Spends time alone
Vigilant	Carefully watches or listens for possible dangers in the surroundings and easily becomes alerted

**Table 4:** Median percent of social interactions for categorical variables assessed during analysis.

Variable	Positive Physical	Positive Non-Physical	Negative Physical	Negative Non-Physical
**Age category**				
Adult	0.60 *	1.57	0.02	0.12 *
Sub-adult	0.50 *	3.56	0.02	0.11 *
Infant	5.99 *	1.46	0.08	0.05 *
Calf	7.40 *	1.16	0.03	0.03 *
**Relatedness to others in herd**				
Related	2.14	2.54 *	0.04	0.07 *
Unrelated	0.11	0.87 *	0.01	0.17 *
**Species**				
African	0.08	0.78 *	0.01	0.13
Asian	2.15	1.77 *	0.03	0.10
**Calf presence**				
Calf present	1.06	2.78 *	0.03	0.11
Calf absent	0.11	0.87 *	0.01	0.10

* indicates significant differences between the categories (*p* < 0.05).
